# Flipping the world upside down: Using eye tracking in virtual reality to study visual search in inverted scenes

**DOI:** 10.16910/jemr.15.3.5

**Published:** 2023-03-31

**Authors:** Julia Beitner, Jason Helbing, Dejan Draschkow, Erwan J. David, Melissa L.-H. Võ

**Affiliations:** Department of Psychology, Goethe University Frankfurt, Germany; Corresponding author, beitner@psych.uni-frankfurt.de; Department of Experimental Psychology, University of Oxford, UK; Oxford Centre for Human Brain Activity, Wellcome Centre for Integrative Neuroimaging, Department of Psychiatry, University of Oxford, UK

**Keywords:** Eye movements, eye tracking, virtual reality, scene perception, visual search, incidental memory, scene inversion

## Abstract

Image inversion is a powerful tool for investigating cognitive mechanisms of visual perception. However, studies have mainly used inversion in paradigms presented on twodimensional computer screens. It remains open whether disruptive effects of inversion also hold true in more naturalistic scenarios. In our study, we used scene inversion in virtual reality in combination with eye tracking to investigate the mechanisms of repeated visual search through three-dimensional immersive indoor scenes. Scene inversion affected all gaze and head measures except fixation durations and saccade amplitudes. Our behavioral results, surprisingly, did not entirely follow as hypothesized: While search efficiency dropped significantly in inverted scenes, participants did not utilize more memory as measured by search time slopes. This indicates that despite the disruption, participants did not try to compensate the increased difficulty by using more memory. Our study highlights the importance of investigating classical experimental paradigms in more naturalistic scenarios to advance research on daily human behavior.

## Introduction

Research of visual perception often employs up-down inversion of visual stimuli as a powerful manipulation to investigate mechanisms of visual processing. The major advantage of image inversion is that it manipulates the whole image, while global image characteristics such as luminance, contrast, and color remain intact (25; 34). Especially the field of face perception has utilized face inversion to probe mechanisms of face recognition (60; 61; 72).

In the study of scene perception, it has been shown that the inversion of scenes has disruptive effects on the top-down extraction of meaning and context (9; 34; 52; 57), specifically on semantic guidance (25). Inversion further affects extraction of scene gist (37; 39), scene categorization (68), scene memory (6; 43), object recognition (39), and eye movement behavior (2; 21).

Scene inversion effects have also been demonstrated for visual search (37). In Koehler and Eckstein’s study, participants had to decide whether a computer mouse was present or absent in two-dimensional images of office scenes. A trial was terminated either after a certain number of fixations (1, 2, or 3) or after 3 seconds, and scenes were either presented upright or inverted on a computer screen. Koehler and Eckstein found that performance dropped when scenes were inverted, that is, overall hit rate was significantly lower for inverted scenes. Eye movement analyses further revealed that the average distance of fixations to the target (if present) or its expected location (in case of absence) was larger for inverted than for upright scenes. Koehler and Eckstein concluded that scene inversion ultimately disrupts guidance in eye movement behavior.

Here, our aim was to test whether these findings generalize to immersive 360-degree visual search and to demonstrate the feasibility of studying scene inversion effects on behavior, including eye movements, in virtual reality (VR). By embracing more unconstrained and naturalistic task settings, VR promises to increase the external validity of findings (16, 17; 26, 27; 50). In some cases, effects demonstrated in laboratory setups were weaker or absent when tested in more realistic settings which often engage behavior that is multimodal, immersive, and self-referential (11; 32; 35; 40; 54; 55, 56; 73). To bridge the gap between the need for high experimental control and naturalistic scenarios, VR offers a promising solution, combining both realism and control (15; 50; 59). Moreover, when comparing VR to experiments in the real world, VR further offers an easy implementation of setups that would physically not be feasible in the real world such as presenting a variety of environments in immediate succession, objects floating in mid-air, participants effortlessly moving objects that would be heavy in reality, or presenting whole environments flipped upside down. Last but not least, one great advantage of tracking eye movements in VR compared to real-world eye tracking is the ability to use the immediately available eye tracking data to directly change the visual input online. This enables interesting new experimental setups such as gaze contingent paradigms in VR (11; 12, 14).

Due to the aforementioned reasons, we decided to investigate the effects of scene inversion on visual search in VR. Previous studies using scene inversion typically found disruptive effects on many levels of visual processing (6; 9; 25; 34; 37; 39; 43; 57). Visual search through scenes is a very efficient process that highly depends on semantic guidance while relying less on episodic memory (8; 12; 18; 27; 63; 64; 65, 66). Scene inversion, however, has been found to actively disrupt this semantic guidance (25; 37).

Since higher task difficulty as well as lack of access to semantic guidance often lead to more memory usage, as indicated by steeper response time slopes across search trials (1; 16; 27; 40; 66), we expected to see worse performance as well as increased memory usage over time (i.e., steeper across-trial search time slopes) in searches through inverted scenes. Using VR in combination with eye tracking allowed a more granular investigation of the eye and head movement dynamics associated with scene inversion effects on visual search. Based on previous research (21; 25), we expected to see more horizontal than vertical saccades and especially a bias for rightward saccades and more head rotations continuously made in the same direction (14) independently of the scene orientation. Given differences in gaze movement distributions, we should further find similar differences in the distributions of head movements as a function of scene orientation since head movements aid eye movements in naturalistic behavior (20; 22; 58; 67). With the vast amount of data generated by VR studies using eye tracking, our analysis of head and eye movements extends beyond our specific hypotheses and takes on an exploratory nature, providing possibly interesting insights for further research in this domain.

## Methods

### Participants

In total, 24 naïve German native speakers were recruited via the university’s recruiting system. Four participants needed to be excluded, i.e., three participants failed to comply with the task instructions, and one participant aborted participation. The final sample consisted of 20 participants (16 women, four men) with a mean age of 22.3 years (*SD* = 3, range = 18–29 years). All participants had normal or corrected-to-normal vision (contact lenses, no glasses), no history of visual or neurological disorders, and were tested for visual acuity (at least 20/25) and normal color vision as assessed by the Ishihara test. All participants volunteered, gave informed consent, and were compensated with course credit or 8 €/h. The experimental procedure conformed to the Declaration of Helsinki and was approved by the local ethics committee of the Faculty of Psychology and Sport Sciences (2014-106R1) at Goethe University Frankfurt.

### Apparatus

During the experiment, participants wore an HTC Vive head-mounted display (HMD) equipped with a Tobii eye tracker and held an HTC Vive controller in their dominant hand. The two 1080 × 1200 px OLED screens inside the HMD have a refresh rate of 90 Hz and a combined field of view of approximately 100° (horizontally) × 110° (vertically). The integrated Tobii eye tracker recorded eye movements binocularly with a refresh rate of 120 Hz and a spatial accuracy below 1.1° within a 20° window centered in the viewports. The experimental procedure was implemented in C# in the Unity 3D game engine (version 2017.3) using SteamVR (version 1.10.26) and Tobii eye-tracking software libraries (version 2.13.3) on a computer operated with Windows 10.

### Stimuli

**Figure 1.**
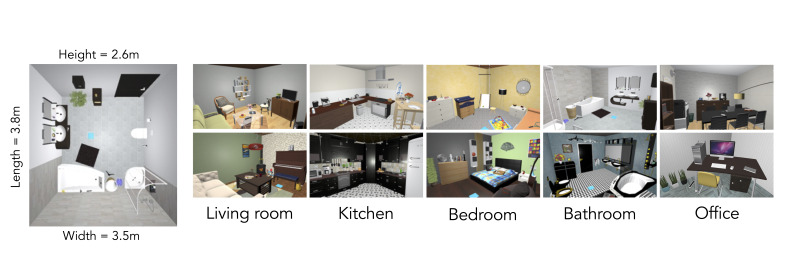
Bird’s-eye view of one of the bathrooms and one sample view of all the scenes that were used in the experiment. Blue squares indicate the starting position of the participants and were not visible during searching.

As in previous studies (7; 11; 12; 27), we used a set of ten in-house developed three-dimensional virtual indoor scenes, two each from five different room categories: bathroom, kitchen, living room, bedroom, and office (see Figure 1). Every scene measured approximately 380 × 350 × 260 cm (length × width × height), which was fitted to the room size of the laboratory where the experiment took place so that participants could naturally move in virtual rooms without fear of colliding with walls. Each scene contained eight global objects which are large, usually static objects (e.g., sink, stove, couch, bed, desk; also known as “anchor” objects, see 8; 18; 27; 63) and 20 local objects, which are smaller objects that are often interacted with (e.g., toothpaste, toilet paper, remote control, alarm clock, keyboard). An additional scene was used for practice trials, which was a gray neutral room including 10 objects which were considered uncommon for typical living indoor spaces (e.g., hydrant, traffic light, diving helmet) to avoid any interference such as priming of any of the succeeding indoor scenes.

### Design

We implemented a repeated visual search task followed by a surprise object recognition task. Critically, we manipulated the inversion of scenes (upright vs. inverted). Upright and inverted scenes were presented randomly interleaved to avoid carry-over effects and the acquisition of strategies over blocks. The condition in which a scene appeared was balanced across participants. Every condition contained five randomly chosen scenes, while each scene was from one of the five unique categories. For each participant individually, 10 out of the 20 local objects were randomly chosen as targets, defining the other 10 as distractors. The search order of objects was randomized. Each scene appeared only once and all 10 target objects were searched in immediate succession. Participants completed a total of 100 searches. The search task was preceded by 10 practice trials in the practice scene, of which five trials were upright and five were inverted. After finishing the visual search task, participants performed a surprise object recognition task to test incidental memory. Results from the object recognition task are not reported here, as eye movements were not recorded and the findings are beyond the scope of the current paper.

### Procedure

Upon entering the lab, participants gave informed consent, performed both vision tests, and were familiarized with the HMD and how to use the controller. Next, the eye tracker was calibrated with a nine-point calibration grid. Participants were then instructed on the visual search task and how to navigate within the environment. Participants were told to search as fast and precisely as possible, and that they could move and walk freely within the virtual room during searching. No information regarding strategies was given. Before entering a scene, participants were presented with an empty gray room with instructions written on a wall. Participants then had to position themselves on a blue square on the floor, which was the starting position for the scene and from where they could see most of the objects without obstructions. When the participants were ready, they pulled the trigger button on the controller in their hand to start the trials. Depending on the condition the scene belonged to, the scene was either upright or inverted. When search trials in a scene started, a fixation cross appeared on a large black square in the center of the participants’ visual field of view for 1 s, followed by a verbal target cue for 1.5 s that informed participants which object to search for (e.g., “Zahnbürste”, toothbrush in German). When the cue disappeared, participants had 15 s to find the target object. Participants completed the trial by pointing a laser beam emerging from their controller at the target and pulling the trigger button with their index finger. In case the selected object was not the target or the timeout was reached, participants heard an error sound. Upon pulling the trigger or after the timeout, the fixation cross cueing the next search appeared. After searching for all 10 target objects successively, participants again entered a gray room with instructions on the wall. The eye tracker was recalibrated after half of the search scenes (i.e., after 50 searches). Participants were allowed to take a break before the eye tracker re-calibration and before the object recognition task if they wanted to. An example video of the task is available at https://osf.io/2ntpj/. Finally, participants completed the object recognition task, which is not reported here. After successful completion of the experiment, participants were debriefed, and exploratively asked how they experienced the different conditions. The whole experiment lasted approximately 1 h. The visual search task reported here lasted approximately 15 minutes.

### Analysis

Only correct trials were included in the analysis of behavior and eye movements measured during the visual search task. We counted cases where a wrong object was selected but gaze was on the target object during selection as correct (5.7%). We chose to perform this correction because participants sometimes had difficulties with aiming the laser beam precisely at the target while pulling the trigger button, resulting in a trial initially being logged as incorrect despite the participant actually having found the target object. All incorrect trials, either where the selected object was not the target or where the timeout was reached, were discarded from further analyses (except for the analysis of search accuracy), which led to the removal of 7.4% of the trials. We further excluded trials in which no saccades were detected (2.15%) or in which the first fixation already landed on the target object (3.55%). This happened when participants already positioned themselves towards the target object while only the cue was visible. Fixations and saccades were determined based on the toolbox of the Salient360! Benchmark (13; 23; 24) using Python (version 3.7.1). To analyze the eye tracking data, we accounted for both head and eye movements and refer to the combination of both as gaze (eye-in-space). By calculating the orthodromic distance and dividing it by the time difference, we obtained the velocity between gaze samples (°/s) which was smoothed with a Savitzky–Golay filter (47). Fixations were identified as filtered samples with a velocity of less than 120 °/s. To further investigate saccade directions, we calculated relative and absolute saccade direction angles based on the analyses described in David et al. (14). Absolute angles are in reference to the longitudinal axis, while relative angles are in reference to the previous saccade angle.

With the preprocessed data, we could further obtain those dependent measures related to visual search (29; 41), that is, initiation time (time until the first saccade was made after cue offset), scanning time (time to first target fixation), and verification time (also known as decision time; after fixating the target, time until button press), as well as gaze durations for each object before it became the target. We excluded trials in which initiation time or verification time exceeded a +3 *SD* cut-off, (4.43%). We analyzed our data using the R statistical programming language (version 4.2.2, 51) with RStudio (version 2022.7.1.554, 53).

To investigate search and eye movement behavior, we calculated generalized linear mixed-effects models (GLMMs) and linear mixed-effects models (LMMs) on all variables of interest using the lme4 package (version 1.1-23, 5). Performing a mixed-models approach allowed us to estimate both between-subject and between-stimulus variance simultaneously which is advantageous compared to traditional F1/F2 analyses of variance (3; 36). We analyzed search accuracy, response time, initiation time, scanning time, verification time, fixation duration (i.e., average duration of all fixations per trial), fixation count, fixated objects count, target refixations (i.e., measure of how many times a participant returned their gaze to the target after a non-target fixation), gaze latitude, saccade amplitudes for gaze and head, as well as gaze and head movement directions.

All variables of interest that were analyzed with LMMs (i.e., response time, initiation time, scanning time, verification time, and fixation duration) were log-transformed to approximate a normal distribution of the residuals and meet assumptions (except gaze latitude, which was left as is). Other variables were analyzed with GLMMs, that is, search accuracy was modeled with a binomial distribution; fixation count, fixated objects count, target refixations, gaze and head directions were modeled using a Poisson distribution; and gaze and head amplitudes were modeled using a Gamma distribution. In all our models, we included search condition (i.e., upright or inverted) as a fixed effect. We added summed gaze durations on the target object from previous trials as a covariate (scaled and centered values) except for the models on gaze and head movement directions because data was averaged across trials. Each model further contained a random effects structure including random intercepts and random slopes for participants and the target object of the respective trial. Note that the models for initiation time, scanning time, verification time, and fixation durations did not include a random slope for participants to avoid a singular model fit (4). To ease understanding of the models’ architecture, we report the model formulas here (1–4) in Wilkinson notation (70). We used difference contrasts to directly compare inverted to upright trials. We obtained *p*-values for LMMs by estimating degrees of freedom with the Satterthwaite method provided by the lmerTest package (version 3.1-2, 38). *p*-values for GLMMs were based on asymptotic Wald tests from the lme4 package (version 1.1-23, 5). All models were fitted with the restricted maximum-likelihood criterion. For each model, we report unstandardized regression coefficients with the *t* or *z* statistic (for LMMs or GLMMs, respectively), and the results of a two-tailed test corresponding to a 5% significance level. Full model results are reported in the online supplementary material, including formula notation and *R*^2^-estimates (28; 45).

Model (1) was used to model search accuracy, fixation count, fixated objects count, target refixations, gaze latitude, gaze and head amplitudes:

**(1):**
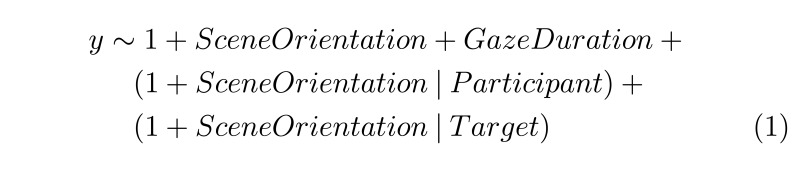


Model (2) was used to model response time, search initiation time, scanning time, verification time and fixation duration:

**(2):**
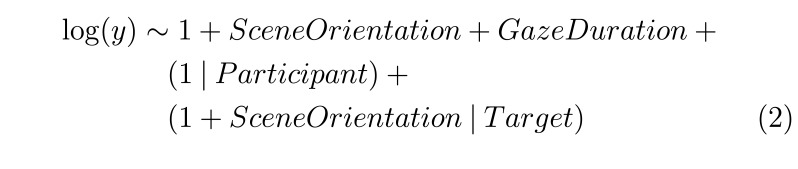


Model (3) was used to model response time across trials and their interaction:

**(3):**
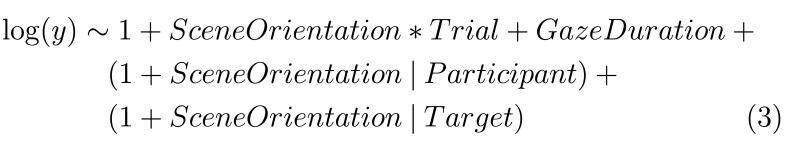


And lastly, model (4) was used to model gaze and head directions:

**(4):**
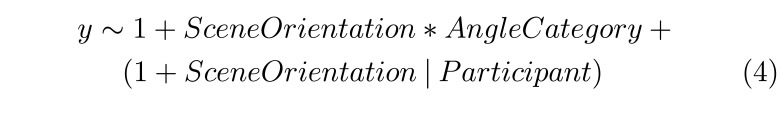


For estimating memory usage over time, we calculated individual slopes across trials on log-transformed response times for both conditions on participant level, and fed them into an LMM with a fixed effects structure including search condition and trial number as well as their interaction and a random effects structure and summed gaze duration as a covariate as mentioned before. We further conducted Bayesian *t*-tests on the participants’ individual slopes to estimate the evidence in favor of a null result. In accordance with Kass and Raftery (33), we interpreted the resulting Bayes factors (BF_01_) as either indicating evidence in favor of the null hypothesis (BF_01_ > 3), or indicating evidence in favor of the alternative hypothesis (BF_01_ < 0.3), or as inconclusive evidence (BF_01_ > 0.3 and BF_01_ < 3). As an example, a BF_01_ = 3 can be interpreted in the sense that the data are three times more likely to stem from the same underlying distribution than from different distributions. Bayesian *t*-tests were computed with the BayesFactor package (version 0.9.12-4.2, 44) and the default settings, that is, with a Cauchy prior with a width of *r* = 0.707 centered on zero. Figures were created with the ggplot2 package in R (version 3.3.0, 69). Dependent measure variable means are reported with their within-subject standard error calculated with the Rmisc package (version 1.5, 30). Data and analysis script can be accessed at https://osf.io/2ntpj/.

## Results

### Behavioral effects

**Figure 2.**
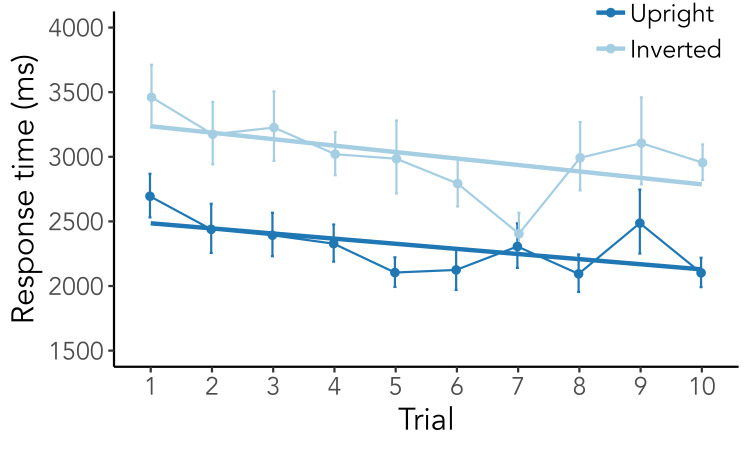
Response times of correct searches from trial 1 to 10 within one scene. Solid straight lines represent regression lines. Solid points indicate means calculated on log-transformed response times which were converted back to their original form for visual purposes, error bars indicate within-subject standard errors.

Participants were descriptively but not significantly less accurate in finding objects in inverted scenes compared to upright scenes (upright: 94.40% ± 3.47%; inverted: 90.80% ± 6.06%; b = −0.81, SE = 0.48, z = −1.71, p = .088). Gaze durations on an object before it became the target object had a significant effect on search accuracy (b = 0.55, SE = 0.18, z = 3.09, p = .002). As can be seen in Figure 2, participants were substantially slower in finding objects when they were searched in inverted scenes (inverted: 3219 ms ± 1417 ms; upright: 2422 ms ± 950 ms; b = 0.31, SE = 0.03, t = 10.42, p < .001), while longer gaze durations again helped in finding objects faster (b = −0.10, SE = 0.01, t = −6.57, p < .001). These findings already indicate that the inversion manipulation posed a higher difficulty to participants.

When looking at memory usage across search trials, we do see a general negative slope across trials (*b* = −0.02, *SE* = 0.01, *t* = −3.00, *p* = .003). This indicates that participants did learn something over time within one scene and used this acquired knowledge to speed their search (see Figure 2). This also fits the finding that gaze duration on objects negatively predicted search times (*b* = −0.08, *SE* = 0.02, *t* = −5.28, *p* < .001). Contrary to our expectations, however, this memory usage did not differ between conditions (*b* = 0.00, *SE* = 0.01, *t* = 0.08, *p* = .934). This result is further supported by the Bayesian *t*-test (BF_01_ = 4.27) indicating that participants used memory to an equal extent regardless of whether they searched through upright or inverted scenes.

### Eye movement measures of search efficiency

**Figure 3.**
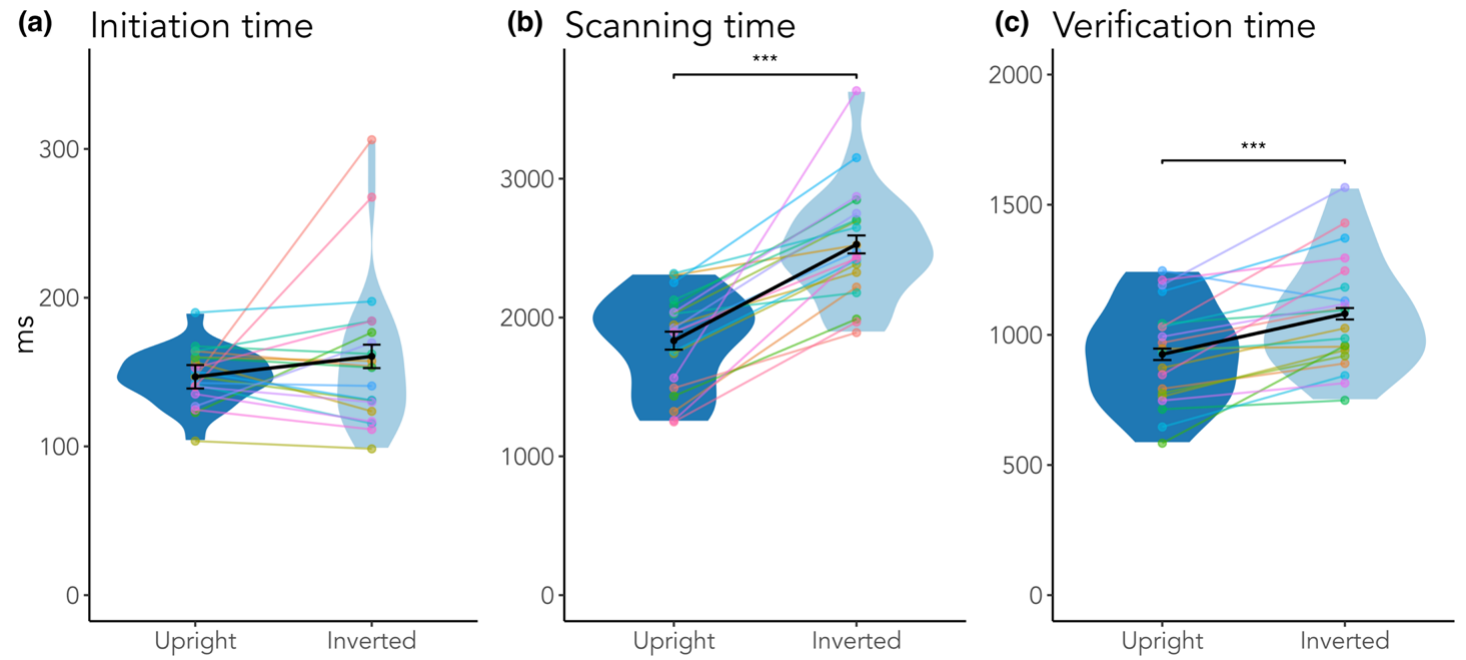
Response time split up into initiation time (a), scanning time (b), and verification time (c). Solid points indicate means, error bars indicate within-subject standard errors, colored points represent individual participants. ***p < .001.

Measuring eye tracking during this immersive task allowed us to decompose the response times into more fine-grained sub-components, that is, initiation time, scanning time, and verification time (29; 41). This procedure enabled us to get a deeper insight into which cognitive processes may be affected by the inversion manipulation. As can be seen in Figure 3 and Table 1, scanning time and verification time were longer in inverted scenes, showing that participants had a harder time finding and recognizing objects. This effect was not present for initiation time.

**Table 1.** Modeling results of eye movement measures of search efficiency.

Effect	Estimate *b* (*SE*)	*t*	*p*
	*Initiation time model*		
Inverted vs. upright	–0.02 (0.06)	–0.37	.715
Gaze duration	0.06 (0.03)	1.96	.050
	*Scanning time model*		
Inverted vs. upright	0.41 (0.05)	8.35	**< .001**
Gaze duration	–0.16 (0.03)	–6.20	**< .001**
	*Verification time model*		
Inverted vs. upright	0.14 (0.02)	5.95	**< .001**
Gaze duration	–0.01 (0.01)	–1.10	.272

Note. Bold p-values indicate significance at an alpha level of 5%.

### Gaze and head measures

**Figure 4.**
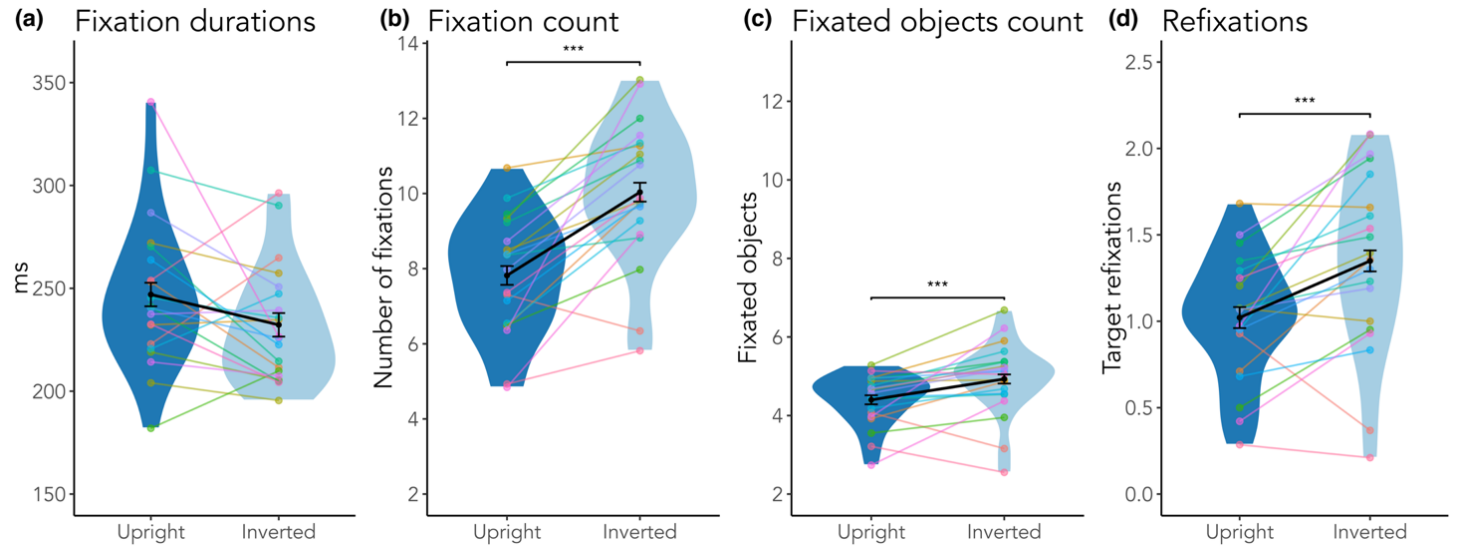
Fixation measures. (a) Fixation durations. (b) Fixation count. (c) Fixated objects count. (d) Refixations. Solid points indicate means, error bars indicate within-subject standard errors, colored points represent individual participants. ***p < .001.

When analyzing fixations, we considered fixation durations, number of fixations within a search trial, the number of objects looked at, and refixations on the target object. While fixation durations did not differ between scene conditions, participants made more fixations in total, fixated more objects on average, and more often refixated the target object during a search trial after having already fixated it previously in inverted scenes (see Figure 4 and Table 2).

**Table 2.** Modeling results of fixation measures.

Effect	Estimate *b* (*SE*)	*t/z*	*p*
	*Fixation duration model*		
Inverted vs. upright	­–0.02 (0.03)	–0.49	.623
Gaze duration	–0.00 (0.01)	–0.13	.898
	*Fixation count model*		
Inverted vs. upright	0.32 (0.05)	6.38	**< .001**
Gaze duration	–0.13 (0.01)	–10.64	**< .001**
	*Fixated objects count model*		
Inverted vs. upright	0.14 (0.04)	3.63	**< .001**
Gaze duration	–0.10 (0.02)	–6.53	**< .001**
	*Target refixation model*		
Inverted vs. upright	0.37 (0.09)	4.02	**< .001**
Gaze duration	–0.24 (0.03)	–6.86	**< .001**

Note. Bold p-values indicate significance on an alpha level of 5%.

**Figure 5.**
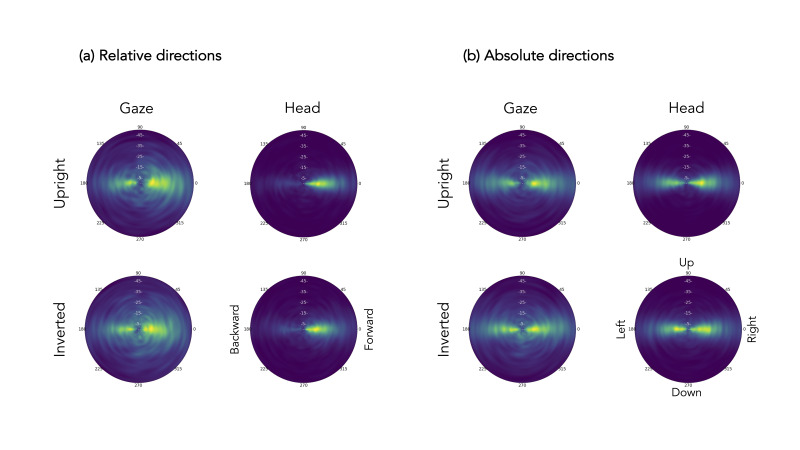
Joint distributions of relative (a) and absolute (b) directions of gaze and head movements and their amplitudes as a function of scene orientation. The lower right plot in each (a) and (b) includes labels for the direction. The lighter the color, the more gaze or head movements were executed into the direction. Radial ticks represent degrees, while ticks from inside out represent saccade amplitudes.

**Figure 6.**
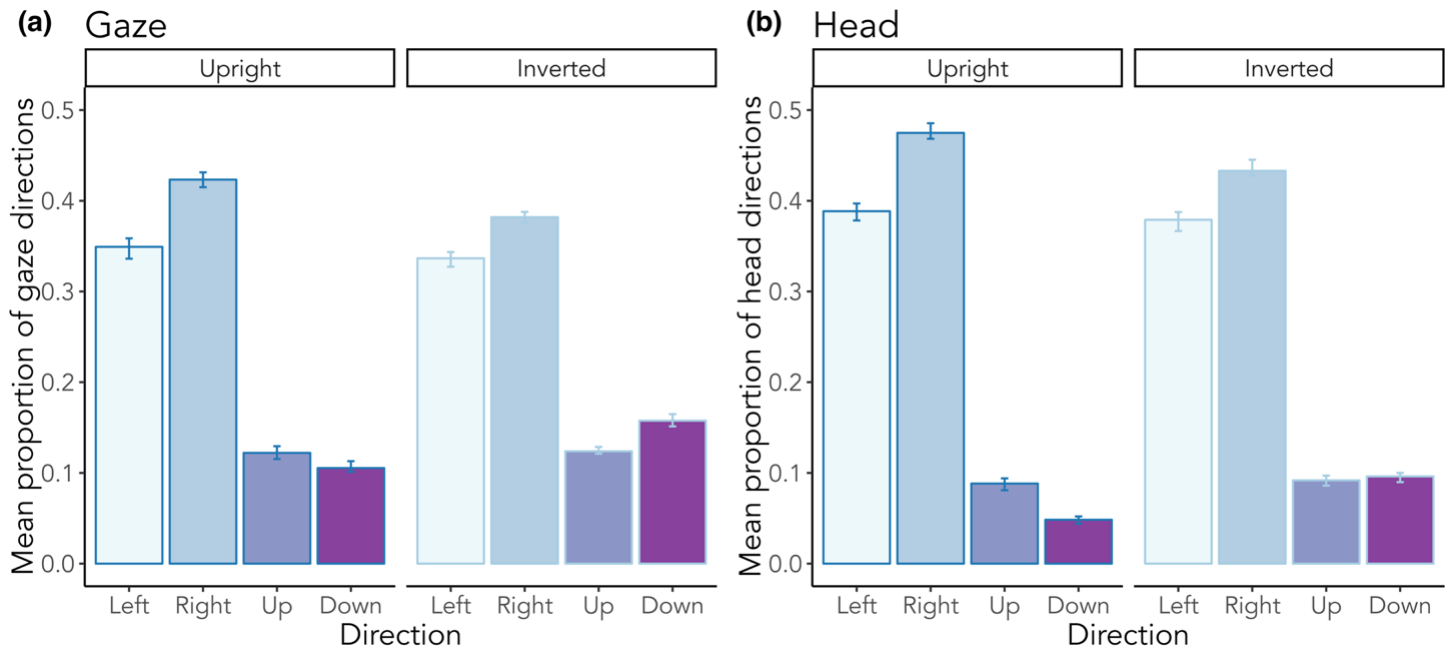
Mean proportion of absolute (a) gaze and (b) head directions as a function of scene orientation. Error bars indicate standard errors.

Looking at saccade directions, we were interested in whether we can observe changes in movement behavior between upright and inverted scenes. First, we looked at the joint distributions of gaze and head movements in relative and absolute angles as described in David et al. (14; see Figure 5). While relative saccade angles are in reference to the previously executed saccade (i.e., a saccade at 0° is a forward saccade moving in the same direction as the previous saccade, while a saccade at 180° can be classified as a backward saccade moving against the direction of the previous saccade), absolute angles are in reference to the longitudinal axis (i.e., 0° is right, 180° is left, 90° is up, and 270° is down). Regarding the relative directions in Figure 5 (a), we see that gaze movements went both forwards and backwards, while head movements were mainly directed forwards. Looking at absolute directions in Figure 5 (b), we see that both gaze and head mainly moved in the horizontal plane as indicated by the lighter green along the horizontal axis. To analyze the data in a quantitative way, we categorized absolute gaze and head directions into left (180°), right (0°), up (90°), and down (270°) with a range of 90° spanning each direction (see Figure 6 2; 14; 21). For example, gaze directions categorized as *left* fell between 135° and 225°.

**Figure 7.**
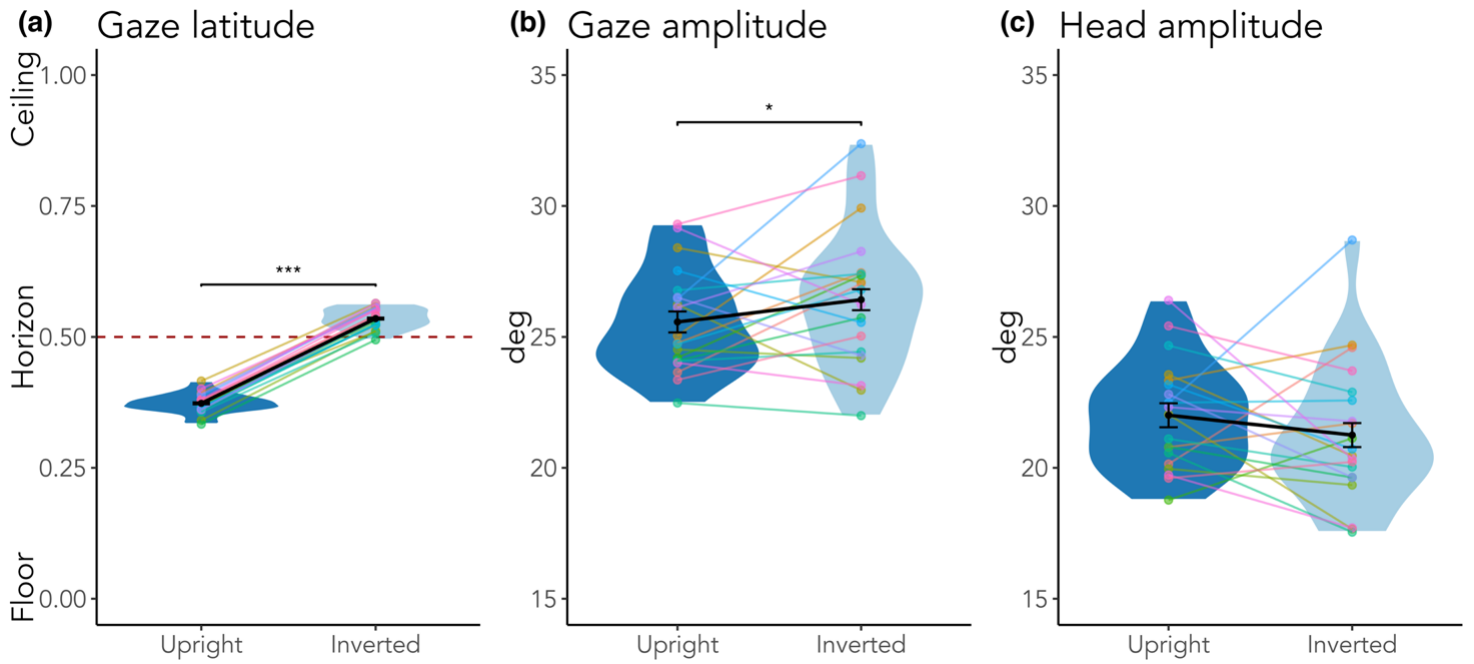
Gaze latitude (a), and amplitudes for (b) gaze and (c) head movements. Solid points indicate means, error bars indicate withinsubject standard errors, colored points represent individual participants. In (a), the dashed red line represents the horizon. *p < .05, ***p < .001.

There was a main effect of scene orientation (*b* = –0.31, *SE* = 0.05, *z* = –6.82, *p* < .001) on gaze direction (Figure 6 a) in that participants moved their gaze more in inverted than upright scenes. We further found a difference between right and left gaze movements (*b* = 0.16, *SE* = 0.02, *z* = 8.47, *p* < .001), indicating that there was a bias for rightward movements. This bias was not significantly present for up and down movements (*b* = 0.05, *SE* = 0.03, *z* = 1.43, *p* = .154). There was a non-significant interaction between right and left movements in upright and inverted scenes (*b* = 0.07, *SE* = 0.04, *z* = 1.75, *p* = .080), and an interaction between up and down movements and scene orientation (*b* = –0.39, *SE* = 0.07, *z* = –5.94, *p* < .001), demonstrating that scene orientation did have an impact on gaze movements in general and especially on up and down movements. These findings basically replicate when looking at isolated head movements (Figure 6 b). Again, more head movements were observed in inverted than in upright scenes (*b* = ­–0.39, *SE* = 0.05, *z* = –8.34, *p* < .001), as well as a bias for rightward movements (*b* = ­0.17, *SE* = 0.02, *z* = 9.41, *p* < .001). In addition, scene inversion again non-significantly interacted with right-left-movements (*b* = 0.07, *SE* = 0.04, *z* = 1.90, *p* = .057) and significantly with up-down-movements (*b* = –­0.65, *SE* = 0.08, *z* = –7.66, *p* < .001). Regarding gaze latitude (i.e., whether participants looked above or below the horizon in relation to them and independent of scene orientation; Figure 7 a), there was a strong difference, showing that participants spent more time looking above the horizon when scenes were inverted (*b* = 0.16, *SE* = 0.01, *t* = 22.83, *p* < .001). Gaze durations on the target did not play a role (*b* = –0.00, *SE* = 0.00, *t* = –0.63, *p* = .530).

Lastly, we investigated amplitudes for gaze and head movements (Figure 7 b and c), and found a small effect of scene inversion on gaze but not head movement amplitudes (*b* = ­–0.07, *SE* = 0.03, *t* = –1.97, *p* = .049; *b* = ­0.03, *SE* = 0.04, *t* = 0.78, *p* = .439, respectively). There was no effect of gaze duration on the target for either amplitudes of gaze or head movements (*b* = ­–0.01, *SE* = 0.01, *t* = –0.46, *p* = .648; *b* = ­–0.01, *SE* = 0.01, *t* = –0.97, *p* = .331, respectively).

## Discussion

With this study, we showed that it is feasible to use scene inversion as a manipulation in VR to investigate cognitive functions such as visual search. In our case, we were interested in whether scene inversion in VR exerts the same disruptive effects on semantic guidance as has been observed in studies using scene inversion in computer-based 2D setups (9; 25; 37). Due to the disruption, we expected to see more memory usage through inverted scenes compared to the usual already very efficient search through upright scenes guided by scene grammar knowledge (8; 12; 18; 27; 63; 64).

In line with the findings by Koehler and Eckstein (37), where search performance dropped in inverted scenes, we similarly found that participants needed more time to find target objects in inverted scenes compared to upright scenes, indicating that scene inversion did impede search efficiency. Splitting up response times into more fine-grained subcomponents of the search process, that is, initiation time, scanning time, and verification time (29; 41), revealed that all phases of the search process except initiation time were negatively affected by inversion. Surprisingly, we did not see increased memory usage during search through inverted scenes, even though search through inverted scenes was more difficult as observed by the overall longer search times. Higher task difficulty is also known to increase memory usage (16). This indicates that participants did not compensate the increased difficulty by using more memory. Instead, semantic guidance—though inverted—might still be accessible to a degree that it can be used to guide search efficiently enough.

In natural behavior there is often little to no need to keep information in visual working memory when the information is still available and only needs to be looked at again (16; 42; 62; 73). Since the information (i.e., location of the target) in our task was still readily available in inverted scenes, participants preferred to simply search again (31) instead of building up more effortful memory using the world as an “outside memory” (10; 48). In both upright and inverted scenes participants could further use their own body movements within the scene to infer spatial relations of object locations (19; 49); information which is lacking in traditional 2D computer screen setups and might lead to different memory encoding strategies (32; 73).

Last but not least, another reason why we did not observe an increase in memory usage might lie in the nature of our VR stimuli. Every scene consisted of eight anchor objects and 20 local objects. However, our VR scenes greatly underestimate the number of objects typically found in real scenes which tend to be much more cluttered (46; 71). Thus, our scenes may have been too sparse to elicit stronger memory effects. This, however, is unlikely to be the determining factor in causing the absence of a memory effect: Other studies using the same (27) or less complex scenes (18) observed strong search slope differences between conditions when other aspects of the scenes were manipulated.

Further, we looked at several gaze measures in an exploratory fashion. In line with Hayes and Henderson (25), we also did not observe an effect on fixation durations between upright and inverted scenes. However, participants made more fixations in total and fixated more objects in inverted scenes, which is likely due to the fact that they also spent more time in total in inverted scenes. In addition, we also observed more target refixations in inverted scenes, which indicates that scene inversion did impede object recognition (34;39). In line with Foulsham and colleagues (21) and Anderson and colleagues (2), we found that most gaze saccades occurred along the horizontal axis regardless of scene orientation. We further found a bias for rightward movements for both gaze and head. In addition, head movements were mainly directed forwards, while gaze also exhibited return saccades (11; 12, 14). When investigating saccade movements, we found that scene inversion affected the pattern of absolute directions of both head and gaze movements, that is, the rightward bias slightly decreased and downward movements increased in inverted scenes.

To summarize, we found that scene inversion in VR did impede search efficiency but did not lead to an uptake in memory usage. Eye tracking allowed us to further investigate gaze and head movement behavior during unrestricted natural behavior and to examine how it was affected by the inversion manipulation. We showed that it is feasible and advances research to use more complex manipulations such as scene inversion in VR in combination with eye tracking to investigate cognitive mechanisms and eye movement behavior in ecologically valid settings. Results can then be compared to studies performed in traditional laboratory setups using 2D computer screens. In our case, we replicated effects of scene inversion, that is, detrimental effects on search performance as well as a change in gaze and head movement directions. However, we could not find the expected increase in memory usage brought about by the disruptions caused by scene inversion. Future studies using within-participant designs might be able to shed more light on how scene inversion affects eye movement behavior and memory usage in 3D environments compared to 2D setups. We hope that our study inspires other vision researchers to implement new paradigms in VR in combination with eye tracking to allow for rigorous and highly controlled investigations of daily human behavior in real-world scenarios.

### Ethics and Conflict of Interest

The authors declare that the contents of the article are in agreement with the ethics described in http://biblio.unibe.ch/portale/elibrary/BOP/jemr/ethics.html and that there is no conflict of interest regarding the publication of this paper.

### Acknowledgements

This work was funded by the Deutsche Forschungsgemeinschaft (DFG, German Research Foundation)—project number 222641018—SFB/TRR 135, sub-project C7 as well as the Hessisches Ministerium für Wissenschaft und Kunst (HMWK), project “The Adaptive Mind” to M.L-H.V., and by the Main-Campus-doctus scholarship of the Stiftung Polytechnische Gesellschaft Frankfurt a. M. to J.B.

The Wellcome Centre for Integrative Neuroimaging is supported by core funding from the Wellcome Trust (203139/Z/16/Z). D.D. is supported by the NIHR Oxford Health Biomedical Research Centre. The funders had no role in the decision to publish or in the preparation of the manuscript.

J.H. is supported by the Deutschlandstipendium scholarship from the Bundesministerium für Bildung und Forschung (BMBF; Federal Ministry of Education and Research) and Goethe University Frankfurt.

We thank Martin Schultze for helpful feedback on the statistical analyses. We would further like to thank Zoë Bolz, Danislava Chuhovska, Aylin Kallmayer, Lea Karoza, and Levi Kumle for help with testing participants.

### Author Contributions

Conceptualization: J.B. & M.L.-H.V.; Methodology: J.B., D.D., M.L.-H.V.; Software: J.B., J.H., E.J.D.; Validation: J.B.; Formal Analysis: J.B.; E.J.D.; Investigation: J.B.; Resources: J.B., J.H., E.J.D.; Data Curation: J.B.; Writing – Original: J.B.; Writing – Review & Editing: J.B., J.H., D.D., E.J.D., M.L.-H.V.; Supervision: D.D., M.L.-H.V., Project Administration: J.B., M.L.-H.V.; Funding Acquisition: M.L.-H.V.

## supplementary material


