# Supplementary material for: Flipping the world upside down: Using eye tracking in virtual reality to study visual search in inverted scenes
Source: J Eye Mov Res. 2023 Mar 31;15(3):10.16910/jemr.15.3.5. doi: 10.16910/jemr.15.3.5 (PMC10195094; doi:10.16910/jemr.15.3.5)
Supplement: Supplementary file 1 [file jemr-15-03-e-SD1-01.pdf]

# Supplementary materials

for Flipping the world upside down: Using eye tracking in virtual reality to study visual search in inverted scenes.

by Beitner, Helbing, Draschkow, David, & Võ (2023)
https://doi.org/10.16910/jemr.15.3.5

Here, we report full results for all linear and generalized linear mixed effects models reported in the manuscript. Result tables and model equations are based on Hoffman and Rovine (2007). Marginal and conditional $R^2$ are estimated using the method implemented in the MuMIn package provided by Nakagawa and Schielzeth (2013).

## Supplementary analysis 1 – Search accuracy

For modeling search accuracy, we calculated a generalized linear mixed effects model using a binomial distribution. To model search accuracy, we applied the following equation (1):

$$\begin{aligned} Level\ 1:\quad & y_{ijk} = \beta_{0jk} + \beta_{1jk}SO_{ijk} + \beta_{2jk}G_{ijk} + \varepsilon_{ijk} \\ Level\ 2:\quad & \beta_{0jk} = \gamma_{00} + U_{0j} + V_{0k} \\ & \beta_{1jk} = \gamma_{10} + U_{1j} + V_{1k} \\ & \beta_{2jk} = \gamma_{20}, \end{aligned} \tag{1}$$

where $y_{ijk}$ is the observed accuracy (0 for incorrect, 1 for correct) and $\varepsilon_{ijk}$ is the residual for target $k$ in search trial $j$ for individual $i$, $SO$ is scene orientation (inverted vs. upright), $G$ is gaze duration on object, and $U$ and $V$ are the random effects for individuals and targets, respectively.

Supplementary table 1. Modeling results of search accuracy.

| | Model Search Accuracy | | |
|---|---|---|---|
| Parameter | Estimate | *SE* | *p* |
| Fixed effects | | | |
| Intercept ($\gamma_{00}$) | 3.57 | 0.26 | **< .001** |
| Scene orientation ($\gamma_{10}$) | –0.08 | 0.48 | .088 |
| Gaze duration ($\gamma_{20}$) | 0.55 | 0.18 | **.002** |
| Variance components | | | |
| Target ($V_{0k}$) | 1.81 | 1.35 | |
| | 1.86 | 1.36 | |
| Participant ($U_{0j}$) | 0.17 | 0.41 | |
| | 0.30 | 0.55 | |
| Fit statistics | | | |
| Pseudo-$R^2_m$; Pseudo-$R^2_c$ | .0670; .4715 | | |
| AIC; BIC | 977.2; 1027.6 | | |

Note. Bold *p*-values indicate significance at an alpha level of 5%.

## Supplementary analysis 2 – Search times

For modeling search times (also called response times), we calculated linear mixed effects models using a Gaussian distribution and log-transformed response times. To model response times, we applied the following equations (2; 3):

$$\begin{aligned} \mathit{Level}\ 1: \quad & y_{ijk} = \beta_{0jk} + \beta_{1jk}SO_{ijk} + \beta_{2jk}G_{ijk} + \varepsilon_{ijk} \\ \mathit{Level}\ 2: \quad & \beta_{0jk} = \gamma_{00} + U_{0j} + V_{0k} \\ & \beta_{1jk} = \gamma_{10} + V_{1k} \\ & \beta_{2jk} = \gamma_{20}, \end{aligned} \tag{2}$$

and

$$\begin{aligned} \mathit{Level}\ 1: \quad & y_{ijk} = \beta_{0jk} + \beta_{1jk}SO_{ijk} + \beta_{2jk}T_{ijk} + \\ & \qquad \beta_{3jk}SO_{ijk} * T_{ijk} + \beta_{4jk}G_{ijk} + \varepsilon_{ijk} \\ \mathit{Level}\ 2: \quad & \beta_{0jk} = \gamma_{00} + U_{0j} + V_{0k} \\ & \beta_{1jk} = \gamma_{10} + U_{1j} + V_{1k} \\ & \beta_{2jk} = \gamma_{20} \\ & \beta_{3jk} = \gamma_{30} \\ & \beta_{4jk} = \gamma_{40}, \end{aligned} \tag{3}$$

where $y_{ijk}$ is the observed log-transformed response time and $\varepsilon_{ijk}$ is the residual for target *k* in search trial *j* for individual *i*, *SO* is scene orientation (inverted vs. upright), *G* is gaze duration on object, *T* is trial number in the scene, and *U* and *V* are the random effects for individuals and targets, respectively.

Supplementary table 2. Modeling results of search times.

| | Model Search Times 1 | | | Model Search Times 2 | | |
|---|---|---|---|---|---|---|
| Parameter | Estimate | *SE* | *p* | Estimate *b* | *SE* | *p* |
| Fixed effects | | | | | | |
| Intercept ($\gamma_{00}$) | 7.88 | 0.04 | **< .001** | 7.96 | 0.05 | **< .001** |
| Scene orientation ($\gamma_{10}$) | 0.31 | 0.03 | **< .001** | 0.30 | 0.06 | **< .001** |
| Trial ($\gamma_{20}$) | | | | –0.02 | 0.01 | **.003** |
| Scene orientation × Trial ($\gamma_{30}$) | | | | 0.00 | 0.01 | .934 |
| Gaze duration ($\gamma_{40}$) | –0.10 | 0.02 | **< .001** | –0.08 | 0.02 | **< .001** |
| Variance components | | | | | | |
| Target ($V_{0k}$) | 0.10 | 0.32 | | 0.10 | 0.32 | |
| | 0.03 | 0.16 | | 0.02 | 0.16 | |
| Participant ($U_{0j}$) | 0.01 | 0.11 | | 0.01 | 0.11 | |
| | | | | 0.00 | 0.52 | |
| Residual variance ($e_{ij}$) | 0.27 | 0.52 | | 0.27 | 0.52 | |
| Fit statistics | | | | | | |
| Pseudo-$R^2_m$; Pseudo-$R^2_c$ | .0709; .3526 | | | .0739; .3592 | | |
| AIC; BIC | 2849.8; 2892.9 | | | 2848.7; 2913.4 | | |

Note. Bold *p*-values indicate significance at an alpha level of 5%.

## Supplementary analysis 3 – Eye movement measures of search efficiency

For modeling initiation time, scanning time, verification time and fixation duration, we calculated a linear mixed effects model using a Gaussian distribution and log-transformed data. We used the following equation (4):

$$\begin{aligned} Level\,1: \quad & y_{ijk} = \beta_{0jk} + \beta_{1jk} SO_{ijk} + \beta_{2jk} G_{ijk} + \varepsilon_{ijk} \\ Level\,2: \quad & \beta_{0jk} = \gamma_{00} + U_{0j} + V_{0k} \\ & \beta_{1jk} = \gamma_{10} + V_{1k} \\ & \beta_{2jk} = \gamma_{20}, \end{aligned} \tag{4}$$

where $y_{ijk}$ is the observed log-transformed time and $\varepsilon_{ijk}$ is the residual for target *k* in search trial *j* for individual *i, SO* is scene orientation (inverted vs. upright), *G* is gaze duration on object, and *U* and *V* are the random effects for individuals and targets, respectively.

Supplementary table 3. Modeling results of initiation time, scanning time, and verification time.

| | Model Initiation Time | | | Model Scanning Time | | | Model Verification Time | | |
|---|---|---|---|---|---|---|---|---|---|
| Parameter | Estimate | *SE* | *p* | Estimate | *SE* | *p* | Estimate | *SE* | *p* |
| Fixed effects | | | | | | | | | |
| Intercept ($\gamma_{00}$) | 4.58 | 0.05 | **< .001** | 7.22 | 0.05 | **< .001** | 6.76 | 0.05 | **< .001** |
| Scene orientation ($\gamma_{10}$) | –0.02 | 0.06 | .715 | 0.42 | 0.05 | **< .001** | 0.14 | 0.02 | **< .001** |
| Gaze duration ($\gamma_{20}$) | 0.62 | 0.03 | .050 | –0.16 | 0.03 | **< .001** | –0.13 | 0.01 | .272 |
| Variance components | | | | | | | | | |
| Target ($V_{0k}$) | 0.03 | 0.17 | | 0.19 | 0.43 | | 0.07 | 0.26 | |
| | 0.06 | 0.25 | | 0.03 | 0.18 | | 0.01 | 0.11 | |
| Participant ($U_{0j}$) | 0.02 | 0.14 | | 0.02 | 0.14 | | 0.04 | 0.20 | |
| Residual variance ($e_{ij}$) | 1.50 | 1.22 | | 0.86 | 0.93 | | 0.17 | 0.42 | |
| Fit statistics | | | | | | | | | |
| Pseudo-$R^2_m$; Pseudo-$R^2_c$ | .0024; .0441 | | | .0535; .2427 | | | .0164; .3922 | | |
| AIC; BIC | 5350.3; 5393.4 | | | 4625.2; 4668.4 | | | 2126.0; 2169.2 | | |

Note. Bold *p*-values indicate significance at an alpha level of 5%.

Supplementary table 4. Modeling results of fixation duration.

| Parameter | Model Fixation Duration | | |
|---|---|---|---|
| | Estimate | *SE* | *p* |
| Fixed effects | | | |
| Intercept ($\gamma_{00}$) | 5.34 | 0.02 | **< .001** |
| Scene orientation ($\gamma_{10}$) | –0.02 | 0.03 | .623 |
| Gaze duration ($\gamma_{20}$) | –0.00 | 0.01 | .898 |
| Variance components | | | |
| Target ($V_{0k}$) | 0.01 | 0.09 | |
| | 0.03 | 0.17 | |
| Participant ($U_{0j}$) | 0.01 | 0.07 | |
| Residual variance ($e_{ij}$) | 0.31 | 0.55 | |
| Fit statistics | | | |
| Pseudo-$R^2_m$; Pseudo-$R^2_c$ | .0002; .0653 | | |
| AIC; BIC | 2785.2; 2828.3 | | |

Note. Bold *p*-values indicate significance at an alpha level of 5%.

## Supplementary analysis 4 – Eye movement count analyses

For modeling fixation count, fixated objects count, and target refixations, we calculated a generalized linear mixed effects model using a Poisson distribution. We used the following equation (5):

$$\begin{aligned} Level\ 1: &\quad y_{ijk} = \beta_{0jk} + \beta_{1jk}SO_{ijk} + \beta_{2jk}G_{ijk} + \varepsilon_{ijk} \\ Level\ 2: &\quad \beta_{0jk} = \gamma_{00} + U_{0j} + V_{0k} \\ &\quad \beta_{1jk} = \gamma_{10} + U_{1j} + V_{1k} \\ &\quad \beta_{2jk} = \gamma_{20}, \end{aligned} \tag{5}$$

where $y_{ijk}$ is the observed count and $\varepsilon_{ijk}$ is the residual for target *k* in search trial *j* for individual *i, SO* is scene orientation (inverted vs. upright), *G* is prior gaze duration on target, and *U* and *V* are the random effects for individuals and targets, respectively.

Supplementary table 5. Modeling results of fixation count, fixated objects count, and target refixations.

| | Model Fixation Count | | | Model Fixated Objects Count | | | Model Target Refixations | | |
|---|---|---|---|---|---|---|---|---|---|
| Parameter | Estimate | *SE* | *p* | Estimate | *SE* | *p* | Estimate | *SE* | *p* |
| Fixed effects | | | | | | | | | |
| Intercept ($\gamma_{00}$) | 2.08 | 0.05 | **< .001** | 1.48 | 0.05 | **< .001** | –0.08 | 0.11 | 0.435 |
| Scene orientation ($\gamma_{10}$) | 0.32 | 0.05 | **< .001** | 0.14 | 0.04 | **< .001** | 0.37 | 0.09 | **< .001** |
| Gaze duration ($\gamma_{20}$) | –0.13 | 0.01 | **< .001** | –0.10 | 0.02 | **< .001** | –0.24 | 0.04 | **< .001** |
| Variance components | | | | | | | | | |
| Target ($V_{0k}$) | 0.16 | 0.40 | | 0.11 | 0.33 | | 0.27 | 0.53 | |
| | 0.18 | 0.42 | | 0.07 | 0.26 | | 0.26 | 0.51 | |
| Participant ($U_{0j}$) | 0.04 | 0.19 | | 0.03 | 0.17 | | 0.19 | 0.43 | |
| | 0.02 | 0.15 | | 0.01 | 0.09 | | 0.08 | 0.27 | |
| Fit statistics | | | | | | | | | |
| Pseudo-$R^2_m$; Pseudo-$R^2_c$ | .0972; .7233 | | | .0345; .4327 | | | .0543; .4103 | | |
| AIC; BIC | 10769.7; 10818.2 | | | 7756.0; 7804.5 | | | 4725.0; 4773.6 | | |

Note. Bold *p*-values indicate significance at an alpha level of 5%.

## Supplementary analysis 5 – Gaze and head directions

For gaze and head directions, we calculated a generalized linear mixed effects model using a Poisson distribution. We used the following equation (6):

$$\begin{aligned} Level\ 1: \quad & y_{ijk} = \beta_{0jk} + \beta_{1jk}SO_{ijk} + \beta_{2jk}AC_{ijk} + \\ & \beta_{3jk}SO_{ijk} * AC_{ijk} + \varepsilon_{ijk} \\ Level\ 2: \quad & \beta_{0jk} = \gamma_{00} + U_{0j} \\ & \beta_{1jk} = \gamma_{10} + U_{1j} \\ & \beta_{2jk} = \gamma_{20} \\ & \beta_{3jk} = \gamma_{30}, \end{aligned} \tag{6}$$

where $y_{ijk}$ is the observed count and $\varepsilon_{ijk}$ is the residual for target *k* in search trial *j* for individual *i, SO* is scene orientation (inverted vs. upright), *AC* is angle category (i.e., right, left, down, and up), and *U* is the random effects for individuals, respectively.

Supplementary table 6. Modeling results of gaze and head directions.

| | Model Gaze Directions | | | Model Head Directions | | |
|---|---|---|---|---|---|---|
| Parameter | Estimate | *SE* | *p* | Estimate | *SE* | *p* |
| Fixed effects | | | | | | |
| Intercept ($\gamma_{00}$) | 4.40 | 0.04 | **< .001** | 4.22 | 0.04 | **< .001** |
| Scene orientation ($\gamma_{10}$) | 0.31 | 0.03 | **< .001** | –0.39 | 0.05 | **< .001** |
| Angle category ($\gamma_{20}$) | | | | | | |
| Right–Left | 0.16 | 0.02 | **< .001** | 0.17 | 0.02 | **< .001** |
| Down–Up | 0.05 | 0.03 | **< .001** | –0.28 | 0.04 | **< .001** |
| Up-Right | –1.19 | 0.03 | .154 | –1.62 | 0.03 | **< .001** |
| Scene orientation × Angle category ($\gamma_{30}$) | | | | | | |
| Orientation×Right–Left | 0.07 | 0.04 | **.080** | 0.07 | 0.04 | .057 |
| Orientation×Down–Up | –0.39 | 0.07 | **< .001** | –0.65 | 0.09 | **<. 001** |
| Orientation×Up–Right | –0.12 | 0.05 | **.026** | –0.13 | 0.06 | **.027** |
| Variance components | | | | | | |
| Participant ($U_{0j}$) | 0.03 | 0.17 | | 0.03 | 0.17 | |
| | 0.03 | 0.18 | | 0.03 | 0.18 | |
| Fit statistics | | | | | | |
| Pseudo-$R^2_m$; Pseudo-$R^2_c$ | .8731; .9733 | | | .9418; .9878 | | |
| AIC; BIC | 1389.5; 1423.3 | | | 1414.9; 1448.7 | | |

Note. Bold *p*-values indicate significance at an alpha level of 5%.

## Supplementary analysis 6 – Gaze latitude, gaze and head amplitudes

For gaze latitude, we calculated a linear mixed effects model using a Gaussian distribution. To model gaze and head amplitudes, we calculated a generalized linear mixed effects model using a Gamma distribution. We used the following equation (7) to model the fixed and random effects structure:

$$\begin{aligned} Level\ 1: \quad & y_{ijk} = \beta_{0jk} + \beta_{1jk} SO_{ijk} + \beta_{2jk} G_{ijk} + \varepsilon_{ijk} \\ Level\ 2: \quad & \beta_{0jk} = \gamma_{00} + U_{0j} + V_{0k} \\ & \beta_{1jk} = \gamma_{10} + U_{1j} + V_{1k} \\ & \beta_{2jk} = \gamma_{20}, \end{aligned} \tag{7}$$

where $y_{ijk}$ is the observed latitude or amplitude and $\varepsilon_{ijk}$ is the residual for target *k* in search trial *j* for individual *i, SO* is scene orientation (inverted vs. upright), *G* is prior gaze duration on target, and *U* and *V* are the random effects for individuals and targets, respectively.

Supplementary table 7. Modeling results of gaze latitude, gaze and head amplitude.

| | Model Gaze Latitude | | | Model Gaze Amplitude | | | Model Head Amplitude | | |
|---|---|---|---|---|---|---|---|---|---|
| Parameter | Estimate | *SE* | *p* | Estimate | *SE* | *p* | Estimate | *SE* | *p* |
| Fixed effects | | | | | | | | | |
| Intercept ($\gamma_{00}$) | 0.45 | 0.01 | **< .001** | 1.45 | 0.03 | **< .001** | 1.57 | 0.03 | **< .001** |
| Scene orientation ($\gamma_{10}$) | 0.16 | 0.01 | **< .001** | –0.07 | 0.03 | **.049** | 0.03 | 0.04 | .439 |
| Gaze duration ($\gamma_{20}$) | –0.00 | 0.00 | .530 | –0.01 | 0.01 | .648 | –0.01 | 0.01 | .331 |
| Variance components | | | | | | | | | |
| Target ($V_{0k}$) | 0.00 | 0.01 | | 0.03 | 0.17 | | 0.07 | 0.26 | |
| | 0.01 | 0.09 | | 0.02 | 0.13 | | 0.02 | 0.14 | |
| Participant ($U_{0j}$) | 0.00 | 0.02 | | 0.01 | 0.09 | | 0.02 | 0.12 | |
| | 0.00 | 0.01 | | 0.01 | 0.09 | | 0.02 | 0.16 | |
| Residual variance ($e_{ij}$) | 0.01 | 0.08 | | 0.95 | 0.97 | | 1.25 | 1.11 | |
| Fit statistics | | | | | | | | | |
| Pseudo-$R^2_m$; Pseudo-$R^2_c$ | .3934; .5711 | | | .0010; .0420 | | | .0000; .0710 | | |
| AIC; BIC | –32047.5; –32000.0 | | | 19974.9; 20051.0 | | | 17832.4; 17909.0 | | |

Note. Bold *p*-values indicate significance at an alpha level of 5%.

## References

Hoffman, L., & Rovine, M. J. (2007). Multilevel models for the experimental psychologist: Foundations and illustrative examples. *Faculty Publications, Department of Psychology*, 421. https://digitalcommons.unl.edu/psychfacpub/421

Nakagawa, S., & Schielzeth, H. (2013). A general and simple method for obtaining $R^2$ from generalized linear mixed-effects models. *Methods in Ecology and Evolution, 4*(2), 133–142. https://doi.org/10.1111/j.2041-210x.2012.00261.x
